# Pattern of pubertal changes in Calabar, South South Nigeria

**DOI:** 10.11604/pamj.2018.31.20.15544

**Published:** 2018-09-07

**Authors:** Michael Eteng Eyong, Happiness Uko Ntia, Joanah Moses Ikobah, Edu Michael Eyong, Helen Uket, Callistus Enyuma, Kelechi Uheagbu

**Affiliations:** 1 Department of Paediatrics, University of Calabar Teaching Hospital, Calabar, Cross River State, Nigeria; 2 Department of Obstetrics and Gynaecology, University of Calabar Teaching Hospital, Calabar, Cross River State, Nigeria

**Keywords:** Puberty, children, secular changes

## Abstract

**Introduction:**

Puberty is an essential physiologic process that is characterized by the appearance of secondary sexual features. Secular changes in puberty occur from one generation to another and need to be documented. The assessment of pubertal stages in a child is only useful if recent and reliable reference data from the same population is available for comparison. The study aimed to profile clinical normative sexual maturity characteristics for children in Calabar, South-South Nigeria.

**Methods:**

A cross-section of primary and secondary school pupils aged 6-18 years in the city of Calabar, Nigeria were randomly selected for the study. Sexual maturating rating was assessed using the pubertal staging for breast development and pubic hair as described by Marshall and Tanner (Tanner staging). Testicular volume in boys was measured using the Prader orchidometer. Menarche and “voice break” were established by recall of the event by the girls and boys respectively.

**Results:**

A total of 2,830 children were seen, 1542 (54.5%) boys and 1288 (45.5%) girls. The mean age of onset of pubic hair was 11 years in both boys and girls. Pubertal testicular volume of 4 mls was seen in boys at a mean age of 11 years. Breast development in girls occurred at mean age of 11 years and menarche at 13 years.

**Conclusion:**

in this study, the onset of puberty is occurring at an earlier age than previously reported in Nigeria with a secular trend of a decreasing age at onset of puberty. These sexual characteristics are rather occurring at similar ages reported from western countries.

## Introduction

Puberty is an important physiologic process that occurs during human growth and development. It marks the transition from childhood to adulthood and the appearance of secondary sexual features is the most visible manifestations of puberty [1]. In boys, testicular enlargement and development of breast buds in girls are the most important early physical signs of puberty and both are as a result of an increased secretion of androgens (testosterone) and estrogen respectively [2]. These increases in gonadal steroids (testosterone and estrogen) secretion is mediated by an increased gonadotropin-releasing hormone secretion from the Hypothalamus which in turn causes an increased secretion of pituitary gonadotropins (Luteinizing and Follicle Stimulating hormones) [2]. The activation of this hypothalamic-pituitary-gonadal axis (gonadarche) as well as maturation of the adrenal axis (adrenarche) leads to normal puberty. Adrenarche is associated with an increase of adrenal androgen production that leads to first appearance of pubic hair. The increases in adrenal androgens secretions often occur prior to increases in pituitary gonadotropins and gonadal steroids (gonadarche) secretion [3]. The progressive physical changes seen are in the breast, genitals and pubic hair. The tanner staging system has divided these continuous physical changes into one of five stages [1, 3]. In boys, there is also a sudden change in voice with ongoing puberty described as “voice break” [4]. In addition to anthropometric changes/secondary sexual characteristics, in boys changes also occur in the organs of phonation. These include an increase in neck length/width and breathing capacity which leads to a relative descent of the larynx and subsequent enlargement of the vocal tract and resonatory system [5]. In natural speech, this voice “break” depends on how the vocal cords vibrate. Spermatogenesis and Menstruation also begins in mid to late puberty [2]. Puberty eventually stops growth in height, at different ages in children through fusion of the epiphysis by the action of estrogens in both boys and girls [6].

Sexual maturation in humans is unique in that there is a 4-5 years physiologic variation in the age at onset of puberty observed among normal individuals living in relatively similar life conditions [7]. Thus, puberty is observed within the age brackets of 8-13 years in girls and 9-14 years in boys [3]. These variability involves genetic factors, ethnicity, nutritional conditions, environmental factors and secular trends in growth and sexual development [7-9]. Secular changes (variation in age at onset of puberty for different generations in a given population) in age of menarche in countries of the Western world have been documented over the last two centuries [8]. A secular trend over these periods has shown a steep decline in age of onset of menarche in the Western world [8, 9]. Secular changes may reflect the varying health and affluence of populations over time and may also be highlighting inequalities of health and wealth within populations [9]. Therefore it is suggested that national reference data should be collected at 10 to 20 years intervals. Furthermore, normative prevalence data on the pubertal characteristics of young girls at various ages are essential for patient education and the provision of appropriate anticipatory guidance [10]. Sexual maturity rating (SMR) especially for those whom growth and development problems are a concern, are only meaningful when suitable reference data in normal healthy peers are available in that population for comparison [9]. There is paucity of literature in South-South Nigeria especially Cross River State, concerning the age at onset of puberty. Normative reference data is often quoted from developed Western countries which may be different from African and Nigerian values. Also, Nigeria is a vast country with different ethnic groups; and growth and puberty has been shown to vary amongst different racial groups [8]. Assessment of secular changes from one generation to the next is impossible without normative reference data to be compared with previous data or in the future [10]. The earlier studies [11, 12] available in literature were carried out about 20 years ago and may not reflect current trends. The aim of this study was to profile the clinical normative pubertal pattern for sexual maturity characteristics of children in Calabar, South-South Nigeria.

## Methods

**Study area:** The study was conducted in Calabar, the capital city of Cross River State, South-South geo-political zone, Nigeria. The residents are mostly from the tribes of Efik, Ibibio, the Quas, Ejagam, and Yakurr people. These tribes are part of the minority tribes that make up Nigeria as distinct from the majority tribes - Hausa (Northern Nigeria), Yorubas (western Nigeria) and Ibos (Eastern Nigeria). There are small number of people from the major tribes also residing in Calabar.

**Study design:** The study was a cross-sectional descriptive study

**Study population:** Children aged between 6-18 years without chronic diseases, growth abnormalities and long term medications like steroids were included for the study. The age of the children studied was calculated based on their last birthday Children of these age groups with chronic diseases, growth abnormalities and on long term medications were excluded from the study. Children who did not give assent to participate in the study as well as those whose parents refused to give consent were also excluded.

**Sampling technique:** Multistage sampling technique was used to recruit subjects for this study. Five Secondary Schools and two Primary schools, in Calabar metropolis were randomly selected and used for the study. A cross section of primary and secondary school pupils aged 6-18 years in the city of Calabar, Nigeria were randomly selected for the study. These public schools draw children from a variety of social and economic backgrounds in the state. The sample size where the population is above 10,000 was estimated from the following formula [13].

$$\text{n=}\frac{{\text{Z}}^{\text{2}}\text{pq}}{{\text{d}}^{\text{2}}}$$

A total of 2401 subjects were recruited for the study.

**Data analysis:** Data was analyzed using EPI info 2002 version. The data is presented in tables with means and standard deviation for continuous variables and as proportions for categorical variables. Percentages were also calculated.

**Ethical consideration:** Ethical Clearance was obtained from the Ethics/Research Committee of the University of Calabar Teaching Hospital, Calabar, Cross River State Ministry of Education, the school headmasters/principals and teachers of all the schools and a written consent was also obtained from the parents of the subjects.

**Data collection:** Sexual maturating rating was assessed using the pubertal staging 1-5, for breast development, pubic hair and genitalia determined by visual inspection as described by Marshall and Tanner (Tanner staging) [1,3]. Testicular volumes in boys were measured using the Prader orchidometer [14]. Menarche was established by recall of the event by the girls. The children were examined in the schools' sick bay or private room that was created for this purpose. The doctors (males and females) that participated in the study were re-trained on measuring testicular volume and determining Tanners staging of puberty. The female doctors examined the girls while the male doctors examined the boys. The bio-data, presence of chronic illnesses, long term medications, and time of onset of menarche was obtained through a questionnaire.

## Results

**General characteristics of the study population:** A total of 2830 children aged 6 to 18 years participated in the study, 54.5% were males and 45.5% were females. Age and sex distribution of the study population is shown in Table 1.

**Table 1:** Study population according to age and sex

Age (years)	Male (%)	Female (%)	Total (%)
6	92	88	180
7	55	40	95
8	53	56	109
9	82	76	158
10	188	196	384
11	256	264	520
12	194	200	394
13	194	134	328
14	144	100	244
15	130	76	206
16	76	40	116
17	46	16	62
18	32	2	34
**Total**	1542(54.5)	1288(45.5)	2830(100)

**Pubertal development (secondary sexual characteristics) in boys**: The distribution of pubic hair in boys according to age is shown in Table 2. The age of onset of pubic hair stage 2 was between 7-15 years. A small proportion of boys had pubic hair development at 7 years with the proportions increasing incrementally with age. The mean age of onset of pubic hair was 11 years (SD 1.27). Adult pattern of pubic hair (stage 5) was seen from 12 years of age. The distribution of testicular sizes according to age is shown in Table 3. Even at the youngest study age of 8 years, a small proportion of boys had pubertal testicular volume of 4ml, with the proportions increasing incrementally with age to 15 years. However, the mean age at testicular volume of 4ml was 11 years (SD 2.18). Testicular volume of 10ml was seen to start at 10 to15 years with a mean age of achieving testicular volume of 10ml at 12.8 years (SD -0.72). Earliest reported age of achieving “voice break” was 10 years as shown on Table 4. The mean age at change in voice to adult type was 13 years.

**Table 2:** Distribution of Pubic hair in boys according to age

Pubic Hair Stage	Age (years)													
	6	7	8	9	10	11	12	13	14	15	16	17	18	**Total**
1	92	49	41	48	86	92	36	20	12	2	-	-	-	478
2	-	6	12	34	80	118	92	46	14	8	-	-	2	412
3	-	-	-	-	22	42	52	58	34	16	6	-	-	230
4	-	-	-	-	-	4	10	62	76	68	32	14	2	268
5	-	-	-	-	-	-	4	8	8	36	38	32	28	154
**Total**	92	55	53	82	188	256	194	194	144	130	76	46	32	1542

**Table 3:** Distribution of testicular size according to age

Age (years)	Testicular Size (ml)												
	1	2	3	4	5	6	8	10	12	15	20	25	**Total**
6	46	46	-	-	-	-	-	-	-	-	-	-	92
7	9	44	2	-	-	-	-	-	-	-	-	-	55
8	5	36	10	2	-	-	-	-	-	-	-	-	53
9	2	58	12	4	6	-	-	-	-	-	-	-	82
10	6	64	52	38	14	6	6	2	-	-	-	-	188
11	2	42	42	66	28	24	24	12	8	6	2	-	256
12	-	24	16	16	30	46	26	6	14	8	6	2	194
13	-	10	10	16	12	24	20	16	24	38	20	4	194
14	-	2	-	10	4	12	10	18	26	34	16	12	144
15	-	-	2	2	2	4	2	14	12	16	50	26	130
16	-	-	-	-	-	-	2	-	6	16	26	26	76
17	-	-	-	-	-	-	-	-	-	6	24	16	46
18	-	-	-	-	-	-	2	-	-	4	12	14	32
**Total**													1542

**Table 4:** Reported voice change in boys according to age

Voice break	Age (years)												
	6	7	8	9	10	11	12	13	14	15	16	17	18
No voice break	92	55	53	82	184	228	152	140	96	56	26	14	16
Voice break present	-	-	-	-	4	28	42	54	48	74	50	32	16
**Total**	92	55	53	82	188	256	194	194	144	130	76	46	32

### Pubertal development (secondary sexual characteristics) in girls

**Pubic hair:** In girls, the development of pubic hair stage 2 was seen as early as 6 years of age as shown on Table 5. However, the mean age of development of pubic hair was 11 years (SD 0.4).

**Table 5:** Distribution of pubic hair development in girls according to age

Pubic hair stage	Age (years)													
	6	7	8	9	10	11	12	13	14	15	16	17	18	**Total**
1	86	36	50	38	46	46	10	6	2	-	-	-	-	320
2	2	4	4	22	76	64	26	2	-	-	-	-	-	200
3	-	-	2	14	48	74	52	30	4	-	-	-	-	224
4	-	-	-	-	26	68	98	78	56	32	10	2	-	370
5	-	-	-	2	-	12	14	18	38	44	30	14	2	174
**Total**	88	40	56	76	196	264	200	134	100	76	40	16	2	1288

**Breast development:** Table 6 shows the distribution of breast development according to age. Breast development stage 2 was seen between ages 8-13 years. The mean age of breast development stage 2 was however 11 years (SD 1.12). Earliest age at development of adult pattern/type breast (stage 5) in study population was 11 years with mean age of 15 years for this stage.

**Table 6:** Breast development in girls according to age

Breast stage	Age (years)													
	6	7	8	9	10	11	12	13	14	15	16	17	18	**Total**
1	88	40	52	40	62	34	6	2						324
2	-	-	4	26	87	96	31	4						248
3	-	-	-	8	37	102	90	32	10					279
4	-	-	-	2	10	22	61	70	36	44	12	4	-	261
5	-	-	-	-	-	10	12	26	54	32	28	12	2	176
**Total**	88	40	56	76	196	264	200	134	100	76	40	16	2	1288

**Menarche:** Table 7 shows age at menarche and corresponding breast development staging. Menarche was reported to occur in two girls at age of 8 years. These two girls had breast development stage 5. Also, 16 girls reported having menses starting at age of 9 years. Two each of these girls had stage 2 and 3 breast development as at time of examining and the rest of the girls, stages 4 and 5. However, the mean age of onset of menstruation was 13 years.

**Table 7:** Age at menarche and corresponding breast development staging

Age	Number achieving menarche	Stage 1 breast	Stage 2 breast	Stage 3 breast	Stage 4 breast	Stage 5 breast
8	2	-	-	-	-	2
9	16	-	2	2	10	2
10	23	-	-	4	9	10
11	61	-	-	17	24	20
12	103	-	2	11	48	42
13	134	-	-	10	64	60
14	52	-	-	4	16	32
15	6	-	-	-	4	2
16	4	-	-	-	2	2
**Total**	401	-	4	48	177	172

## Discussion

In boys, the development of pubic hair in this study occurred at a mean age of 11 years. A small proportion of boys developed pubic hair at 7 years with the proportions increasing incrementally with age. Adult pattern pubic hair was seen from 12 years of age. This finding is in contrast to one previous report in Nigeria by Ezeome et al [11] where pubic hair development was reported to occur from 9-15 years. It is however, similar to reports from United States of America and the Netherland where pubic hair development was seen as early as 7 and 8 years respectively [10, 15]. The mean age of pubic hair development of 9 years in this study was also similar to the report from the United States of 8.78 years in African-American girls but lower than the 10.51 years reported on American white girls [10]. Pubic hair development on its own may not be a sign of onset of puberty as it may represent early surge of Adrenal androgen release (Adrenarche) [2, 3]. Therefore using it by previous Nigerian authors to signify onset of puberty in boys can be misleading. The most reliable sign of onset of puberty is the testicular enlargement. [3, 14] However, because both the activation of the hypothalamic-pituitary-gonadal axis (gonadarche) as well as maturation of the adrenal axis (adrenarche) is needed for normal puberty, the appearance of pubic hair remains an important landmark in describing puberty especially to parents and the public. Onset of puberty in boys is said to start when testicular volume reaches 4 mls by the Prader orchidometer [3, 14]. This is an easy parameter to assess in boys in the outpatient clinic and during research. A small proportion of boys at 8 years had testicular volume of 4 mls and these proportions increased with age. This age of pubertal onset of testicular volume >4 mls in boys in this study is occurring one year lower than the accepted norm of 9 years [3]. However, the mean age of testicular volume of 4 mls attainment was 11 years and compares with report from Netherland of 11.5 years [15]. There is no other work to our knowledge on testicular volume in boys using Prader orchidometer in this part of South-South Nigeria to compare. However, it would appear that the attainment of puberty in boys in Nigeria is similar to that seen in Europe and the Western world where puberty is said to start from 9-14 years [3]. This may be due to an improved economy since 1999 translating to improved living standard and good health compared to previous decades in Nigeria [16, 17]. This also reflects a positive secular change towards early puberty in boys in Nigeria compared to previous report by Ellis [12] about 6 decades ago where pubic hair was noticed from 10 years and genital development from 12 years.

This earlier study used a small sample size of 333 boys and is prone to errors as genital development may be difficult to assess in black skin children and testicular volume was not used in the study. The mean age of voice change ("voice break") to adult type in this study was at 15 years and may correspond to testicular volume of at least 15 mls. This result is similar to that of earlier report of 14 years in the United Kingdom [5]. There are no previous studies of voice “break” in Nigeria to my knowledge. Even though it is a recall event in this study, the result is still reliable as the event is not far from the subject's ages and they can recall it. In girls, the development of secondary sexual characteristics in this study is similarly occurring at younger ages than suggested in previous reports from Nigeria [12, 18], Senegal and Ethiopia [19, 20]. On the average, girls in this study begin pubertal development between the ages of 8 to 13 years as is in keeping with internationally accepted norm [3]. Breast development was seen in a small proportion of girls at 8 years and increase in proportions with age. The mean age of breast development was 11 years. This is in contrast to earlier reports of mean ages of 12.7 years in Nigeria [18] and 12.6 years in Senegal [19]. In this study, girls appear to be developing breast 6 months-1.5 years sooner than in previous reports [12, 19] and rather similar to their European counterpart of 10.7 years in Netherlands [15] and 9.96 years in white American girls [10]. This may still be due to improved living conditions and good health experienced in Nigeria over the past decades [16, 17]. The development of pubic hair in girls in this study was similar to that seen in boys and was noticed as early as 6 years with a mean age of 11 years. A similar trend has also been reported in African American girls as young as 6 years developing pubic hair but with a mean age of 8.78 years [10]. The mean age of pubic hair growth of 11 years is similar to that of 10.51 years for white American girls [10].

In this study, menarche was reported to occur in two girls at the age of 8 years. The corresponding breast development at the time of this study was stage 5 for these 8 year olds (which may have been lower at the time of onset of the menses). Sixteen girls also reported onset of menses at the age of 9 years. Four of these girls had stage 4 breast development and 12 of them had stages 5. This age of onset of menarche is similar to that reported in African American girls at 9 years in an American study [10]. It may also be that these girls just had precocious puberty with breast development occurring earlier than 8 years of age but now culminating in puberty at this age of 8 and 9 years. Also, age at menarche is said to be more sensitive to nutritional and chronic infection status [10, 21, 22]. It is possible, better nutrition and health experienced by children in most urban Nigeria towns recently may account for this early onset of menstruation and puberty [16]. Ellis [12] observed six decades ago, a mean age of menarche in two series of studies in Nigerian girls to be 14.22 and 14.44 years. Even though the sample size was small (300 girls in the 1^st^ series and 250 girls in 2^nd^ series), the result may still reflect the pubertal changes occurring then in Nigeria. The mean age of menarche in this study was 13 years. As age at menarche were asked as ages in whole years, the data about age at menarche may not be very precise. However, it will appear from this study that there is a positive secular change towards a decreasing age at menarche/puberty by about 1.5 years. This is similar to the reports from Europe over the decades [8, 23]. This result of age at menarche of 13 years is also similar to a more recent study in the 1990s from Nigeria with reported mean age at menarche of 13.6 and 13.9 years [19, 24] and a more current report of 13.02 years [25] in 2010. It would appear that there is an apparent stabilization of age at menarche at 13 years in Nigeria. It is interesting to note that this age at onset of menarche is similar to that reported from America (12.5 years) [10], Netherland (13.5 years) [15], Uganda (13.3 years) [22] and Bangladesh (13 years) [21]. On the other hand, the result is lower than that reported from some other African countries, Ethiopia (14.8 years) [20] and Senegal (15.9 years) [**19**]. These two African studies used adolescents in rural communities where living conditions were said to be poor and nutrition not optimal.

## Conclusion

There is a downward age trend in start of pubertal development in both boys and girls in Nigeria. It is recommended that regional studies be carried out to corroborate these findings.

### What is known about this topic

- Puberty is a known physiologic change that occurs in children as they develop into adults with unique physical characteristics: the onset of these pubertal characteristics is well documented in the Western countries and America;
- There is also a documented secular change over the decades regarding a decreasing age at onset of puberty in these countries; there is paucity of literature on this important health issue in Nigeria especially in the South- South of Nigeria.

### What this study adds

- Unlike most studies done in Nigeria and Africa that focused mostly on onset of menarche in girls, this study looked at all the secondary sexual characteristics in both boys and girls (pubic hair growth, breast development, voice change, testicular enlargement and menarche);
- The onset of puberty (breast development) for girls in this study was from 8 years to 13 years with a mean age of 11 years: this is in agreement with the international definition of onset of puberty by endocrinologists; the onset of Puberty for boys is not determined by the development of pubic hair which previous authors erroneously used; testicular enlargement of > 4 mls by prader orchidometer is internationally recognised in clinical practice and in research; in this study, it occurred from 8 years to 15 years with a mean age of 11 years;
- This study shows that puberty begins at eight years and so policy makers in government should incorporate education on puberty and sexuality in the school curriculum from this age.

## Competing interests

The authors declare no competing interest.

## References

[1] Marshall WA, Tanner JM (1970). Variations in the pattern of pubertal Changes in Boys. Arch Dis child.

[2] Lee PA, Houk CP, Lifshitz F (2007). Paediatric Endocrinology.

[3] Rodriguez H, Pescovitz OH, Radovick S, MacGillivray MH (2003). Paediatric Endocrinology: a practical clinical guide, Precocious Puberty.

[4] Marshall WA, Tanner JM (1969). Variations in the pattern of pubertal changes in girls. Arch Dis Child.

[5] Harries MLL, Walker JM, Williams DM, Hawkins S, Hughes IA (1997). Changes in the male voice at puberty. Arch Dis child.

[6] Gelander L (1998). Growth in prepubertal children-short term changes and endocrine regulation. the one year growth study. Coteborg.

[7] Parent A, Teilmann G, Juul A, Skakkebaek NE, Toppari J, Bourguignon J (2003). The timing of normal puberty and the age limits of sexual precocity: variations around the World, Secular Trends and changes after Migration. Endocrine Reviews.

[8] Cole TJ (2000). Secular trends in growth. Proceedings of the Nutrition Society.

[9] Brook CGD, Brown RS, Brook CGD, Brown RS (2008). Problems of puberty and Adolescence. Handbook of clinical Paediatric Endocrinology.

[10] Herman-Giddens ME, Slora EJ, Wasserman RC, Bourdony CJ, Bhapkar MV, Koch GG, Hasemeier CM (1997). Secondary Sexual characteristics and Menses in young Girls seen in Office practice: a study from the Paediatric Research in Office settings network. Paediatrics.

[11] Ezeome ER, Ekenze SO, Obanye RO, Onyeagocha AC, Adibe LN, Chigbo J, Onuigbo WI (1997). Normal pattern of pubertal changes in Nigerian boys. West Africa J Med.

[12] Ellis RWB (1950). Age at puberty in the tropics. Brit med J.

[13] Araoye MO, Araole MO (2004). Research methodology with statistics for health and social sciences.

[14] Karaman MI, Kaya C, Guney TCS, Ergenekon E (2005). Measurement of Paediatric testicular volume with Prader Orchidometer: comparison of different hands. Pediatr Surg int.

[15] Mul D, Frediks AM, Van Buuren S, Oosdijk W, Werloove-Vanhorick SP, Wit JM (2001). Pubertal Development in the Netherlands 1965-1997. Paediatri Research.

[16] Olayiwola K, Soyibo A, Atinmo T (2003). Impact of globalization on food consumption, health and nutrition in Nigeria: a report on Globalization of food systems in developing countries. Impact on food security and nutrition.

[17] Kelikume M (2015). Economic Development and Growth in Nigeria. Daily Trust Newspaper.

[18] Fakeye O, Fagbule D (1990). Age and anthropometric status of Nigerian girls at puberty: implication for the introduction of sex education into secondary schools. West Afr J med.

[19] Garnier D, Simondon KBDE, Neafice E (2005). Longitudinal Estimates of Puberty Timing in Senegalese Adolescent Girls. Am J Human Biol.

[20] Zegeye DT, Megabiaw B, Mulu A (2009). Age at menarche and the menstrual pattern of secondary school adolescents in northwest Ethiopia. BMC Women's health.

[21] Chowdhury S, Shahabuddin AKM, Seal AJ, Talukder KK, Hassan Q, Begum RA, Rahman Q, Tomkins A, Costella A, Talukder MQK (2000). Nutritional status and age at menarche in a rural area of Bangladesh. Annals of Hum Biol.

[22] Odongkara MB, Piloya T, Awor S, Ngwiri T, Laigong P, Nworozi EA, Hochberg Z (2014). Age at Menarche in relation to Nutritional Status and critical life events among rural and urban secondary school girls in post conflict Northern Uganda. BMC women's health.

[23] Keizer-Schrama SMPFM, Mul D (2001). Trends in Pubertal Development in Europe. Human Reproduction update.

[24] Abioye-Kuteyi EA, Ojofeitimi EO, Aina OI, Kio F, Aluko Y, Mosuro O (1997). The Influence of Socioeconomic and Nutritional Status on Menarche in Nigerian School Girls. Nutr Health.

[25] Goon DT, Toriola AL, Uever J, Wuam S, Toriola OM (2010). Growth Status and Menarcheal Age among Adolescent School Girls in Wannune, Benue State, Nigeria. BMC Paediatrics.

